# Fractal fluctuations in exploratory movements predict differences in dynamic touch capabilities between children with Attention-Deficit Hyperactivity Disorder and typical development

**DOI:** 10.1371/journal.pone.0217200

**Published:** 2019-05-21

**Authors:** Bruna S. Avelar, Marisa C. Mancini, Sergio T. Fonseca, Damian G. Kelty-Stephen, Débora M. de Miranda, Marco Aurélio Romano-Silva, Priscila A. de Araújo, Paula L. Silva

**Affiliations:** 1 Graduate Program in Rehabilitation Science, School of Physical Education, Physical Therapy, and Occupational Therapy, Universidade Federal de Minas Gerais (UFMG), Belo Horizonte, Minas Gerais, Brazil; 2 Department of Psychology, Grinnell College, Grinnell, Iowa, United States of America; 3 Department of Pediatrics, School of Medicine, UFMG, Belo Horizonte, Minas Gerais, Brazil; 4 Department of Mental Health, School of Medicine, UFMG, Belo Horizonte, Minas Gerais, Brazil; 5 Center for Cognition, Action & Perception, Department of Psychology, University of Cincinnati, Cincinnati, Ohio, United States of America; Massachusetts General Hospital, UNITED STATES

## Abstract

Children with Attention-Deficit Hyperactivity Disorder (ADHD) struggle to perform a host of daily activities. Many of these involve forceful interaction with objects and thus implicate dynamic touch. Therefore, deficits in dynamic touch could underlie functional difficulties presented by ADHD children. We investigated whether performance on a dynamic touch task (length perception by wielding) differ between children with ADHD and age-matched controls. We further examined whether this difference could be explained by fractal temporal correlations (wielding dynamics). Forty-two children (ADHD: 21; typically developing: 21) wielded unseen wooden rods and reported their perceived length in the form of magnitude productions. The rods varied in the magnitude of the first principal moment of inertia (*I*_1_). Three-dimensional displacements of hand and rod positions were submitted to Detrended Fluctuation Analysis to estimate trial-by-trial temporal correlations. Children with ADHD reported shorter length for rods with higher *I*_1_ than their typically developing peers, indicative of reduced sensitivity to mechanical information supporting dynamic touch. Importantly, temporal correlations in wielding dynamics moderated children’s usage of *I*_1._ This finding points to a role of exploratory movements in perceptual deficits presented by children with ADHD and, thus, should be considered a new potential target for interventions.

## Introduction

Attention-Deficit Hyperactivity Disorder (ADHD) is characterized by symptoms of inattention and/or hyperactivity-impulsivity associated with reduced independence at home and deficient academic performance at school [1–3]. In addition to functional limitations associated with cognitive behavioral symptoms, children with ADHD also have difficulties with the performance of perceptual-motor activities, which are key for successful functioning at home and school settings [4]. The present study aimed to determine whether perceptual deficits might be related to difficulties these children have to move objects in ways that promote learning about their properties and effective use.

Performance deficits in children with ADHD commonly appear in the context of daily activities that involve interaction with objects, such as silverware, scissors, tweezers, toothbrush, pencil, or ball [2,5–7]. To perform these activities successfully, children have to carefully coordinate their muscular forces to manipulate the object in a way that meets the activity requirements. This careful patterning requires keen perception of the object’s geometrical and dynamical properties [8,9], relying on a haptic perceptual subsystem called dynamic touch [9,10,11]. Perception by dynamic touch in turn requires effortful engagement with objects. Therefore, the difficulty in performing motor activities presented by children with ADHD may produce deficits in dynamic touch and vice-versa.

Dynamic touch supports accurate and reliable perception of a number of object properties, such as length [8,12–14], width [13], heaviness [15], and center of percussion [16]. Manipulations of the mechanical properties of the hand-held objects have allowed researchers to consistently conclude that the basis for these accomplishments is the invariant resistance offered by hand-held objects to their motion (i.e., the objects’ mass moments). Deficits in dynamic touch can, thus, result when a pathological condition compromises processes that would typically guarantee sensitivity to objects’ mass moments.

To appreciate how an object’s mass moments might constrain perception by dynamic touch, consider the well-studied task of perceiving the length of hand-held rods by wielding. The first principal moment of inertia (*I*_1_) of rods constructed of the same homogenous materials relate reliably to their length. If one is to hold a rod firmly at its proximal end while pointing it forward, *I*_1_ will be related to the resistance the rod will offer to up-down and side-to-side accelerations [8,12]. Therefore, wielding the rod in these directions can create the mechanical stimulation that supports detection of *I*_1_ and, thus, perception of rod length. When the task is performed successfully, participants exploit the lawful relationship that exist between *I*_1_ and the length of homogenous rods—Length = a (*I*_1_)^b^, with b = .33 as expected from dimensional analysis.

The perception of length by wielding has been the paradigm of choice to study dynamic touch in children with and without developmental disability. Research has shown that perceptual performance improves with age [17,18], with typically developing (TD) children reaching adult performance between eight and ten years old [19]. Notably, children with clinical conditions, such as cerebral palsy and developmental coordination disorder (DCD), had lower accuracy in perceptual reports than TD children and reduced attunement to *I*_1_ [19,20]. It is unknown, however, whether individuals with ADHD show similar deficits. If they do, remediation requires an understanding of what might be limiting the appropriate use of mechanical information.

Understanding inter-individual differences in the use of mechanical information requires focusing on exploratory movements (wielding dynamics). There are multiple means to explore an object to successfully perceive its properties (e.g. moving the wrist, the elbow, and the shoulder or simply holding the object yield similar perceptual reports) [21,22]. Thus, traditional kinematic descriptions of exploratory movements cannot distinguish their effectiveness. Research has shown, however, that if we remove the overall trend in exploratory movements (overt or more static), the long-range correlations of left-over fluctuations (sometimes called “fractality”) predict interindividual differences in perceptual performance [23,24,25–27].

Fractal fluctuations in haptic exploration prove to be critical for perceptual learning. They reflect the coordination of sampling information across various times scales: (a) detection in short-term information pick-up; (b) calibration with medium-term rescaling judgments of object properties and (c) attunement at the longer-term as participants change which object variable they attend to [28,29]. Crucial for present purposes, research showed that wielding dynamics (i.e., long-range temporal correlations in exploratory movements) predicts how an individual uses information in dynamic touch tasks on a trial-by-trial basis and, thus, explains inter-individual differences in perceptual performance and perceptual learning [23,24,25–27]—even in the case of non-overt postural sway [30].

We proposed to investigate the performance of children with ADHD in dynamic touch and examined whether fractal measures of their exploratory movements explain their differences in dynamic touch from TD children. Initially, we might well anticipate that the perceptual-motor difficulties and, indeed, difficulties following rules [31] could lead children with ADHD to struggle to meet the demands of the basic dynamic touch procedure, e.g., maintaining a firm grasp on the wielded object. Hence, we tracked exploratory movements with motion-capture both on the hand, in accordance with past dynamic-touch research, but also on the rod itself. This elaboration of the standard method would not only anticipate the failure of children with ADHD to maintain firm grasp according to the experimental instructions, but it would also have the added benefit of elaborating early evidence that incidental vibrations of the rod itself provide perceptual information above and beyond wielding behaviors [32,33]. For instance, it may be that the fractal structure of rod vibrations offers a competing source of information, and children with ADHD might even use this source for their dynamic-touch reports. We hypothesized that (1) children with ADHD would be less accurate and reliable in the length perception task compared to TD children, and (2) differences in performance would be explained by reduced reliance on *I*_1_, which in turn would be associated with the fractal structure of wielding dynamics.

## Materials and methods

### Participants

The ethics review committee of the Universidade Federal de Minas Gerais (UFMG, Brazil) approved the study. In compliance with the approved research protocol, informed consent was obtained, in writing, from all participants and their caregivers prior to their inclusion in the study.

Initially, 24 children with diagnosis of ADHD established according to the Diagnostic and Statistical Manual of Mental Disorders (DSM-5 [1]) composed the ADHD group and 22 TD children with no history of any pathology or clinical disorder composed the TD group. Specifically, children with ADHD and their parents underwent a semi-structured psychiatric diagnostic interview with the Brazilian version of the Schedule for Affective Disorders and Schizophrenia for School-Age Children-Present and Lifetime Version (K-SADS-PL). These children were recruited through convenience sampling from the child psychiatry outpatient clinic at Hospital das Clínicas /Universidade Federal de Minas Gerais (UFMG). TD children were recruited from the local community. Parents of children with ADHD agreed not to administer medication (psychostimulants) for 24 hours prior to their experimental section. UFMG’s ethics review committee approved the study.

All children initially selected to participate in the study were submitted to the Raven’s Colored Progressive Matrices (Raven’s CP; [34]). The Raven’s CP was used to document children’s cognitive ability. Only children with average intellectual ability, that is, those able to understand and cooperate with verbal instructions, continued in the study (Table 1). Three children with ADHD were excluded due to their scores on the Raven’s CP. Parents from both groups completed the Brazilian version of the Swanson, Nolan, and Pelham Questionnaire—version IV (SNAP-IV), a behavior rating scale used to assess symptoms of ADHD (subscales: inattention and hyperactivity-impulsivity; [35]). Following the diagnostic criteria established by the DSM-V, only children who had at least six items rated "quite a bit" or "very much" in one or more subscales of SNAP-IV were included in the ADHD group. Only children who had less than six items rated "quite a bit" or "very much" in any subscale of SNAP-IV were included in TD group. At this stage, one TD child was excluded and referred for medical evaluation. Table 1 provides a detailed characterization of each group.

**Table 1:** Description of ADHD and TD groups.

Characteristics		Group		*p* value^f^
		ADHD (n = 21)	TD (n = 21)	
Sexᵃ	Male	18 (86%)	15 (71%)	0.259
	Female	3 (14%)	6 (29%)	
Ageᵇ	Years	9.9 (1.3)	10.2 (1.5)	0.400
Body Weightᵇ	Kilograms	39 (9.2)	40.2 (10.2)	0.691
Heightᵇ	Centimeters	145 (10)	145.6 (10.1)	0.842
Cognitive Functionᵇ	Raven's CP	76 (24.7)ᶜ	80.6 (21.6)ᶜ	0.603
Socioeconomic statusᵃ	CCEB categoriesᵈ	A = 3 (14%)	A = 3 (14%)	
		B1/B2 = 8 (38%)	B1/B2 = 8 (38%)	
		C1/C2 = 10 (48%)	C1/C2 = 10 (48%)	
		D/E = 0	D/E = 0	
ADHD behaviorsᵃ	SNAP—IV^e^			
	Inattentive	5 (24%)	N/A	
	Hyperactive-impulsive	6 (29%)	N/A	
	Combined	10 (47%)	N/A	
Preferred limbᵃ	Right	21 (100%)	21 (100%)	

ADHD: Attention-Deficit Hyperactivity Disorder, TD: typically developing, Raven’s CP: Raven’s Colored Progressive Matrices SNAP—IV: Brazilian version of the Swanson, Nolan, and Pelham—version IV, N/A: not applicable.

ᵃ Numbers indicate frequency and (%).

ᵇ Numbers indicate mean and (standard deviation).

ᶜ Scores within or above the “average” descriptive category, which range from 26 to 99 (R.C. Raven 1999).

ᵈ CCEB Brazil’s socioeconomic classification criteria. The categories represent family’s socioeconomic levels. They are defined from a standardized questionnaire that assigns points to items related to the presence and amount of certain home appliances, number of cars owned, and the level of formal education of the main family member (provider). The points are summed, and specific ranges are translated into categories, in which higher total scores refer to higher socioeconomic levels. Level A = 45 to 100 points; B1/B2 = 29 to 44 points; C1/C2 = 17 to 28 points; and D/E = 0 to16 points.

^e^ SNAP-IV: Inattentive = at least six items symptoms rated "quite a bit" and "very much" in inattention; Hyperactive-impulsive = at least six items symptoms rated "quite a bit" and "very much" in hyperactive-impulsive; combined = Inattentive + Hyperactive-impulsive.

^f^ Equivalence between groups was verified by independent-sample T test.

### Experimental procedures

**Fig 1:**
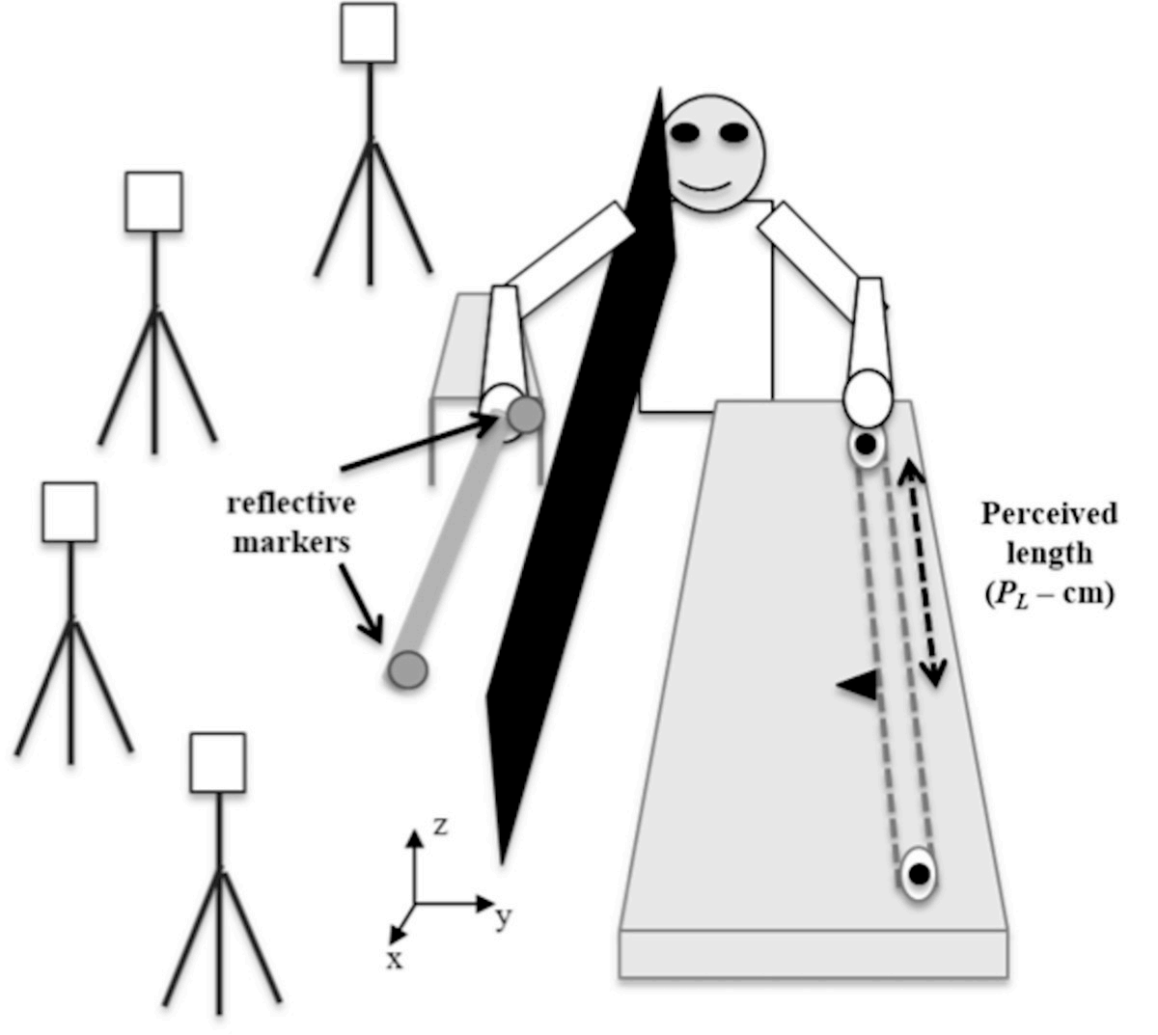
Experimental set up: Occluded rods were wielded by hand as participants adjusted a visible marker to indicate the location of the tip. Four ProReflex cameras recorded the motions of reflexive markers positioned at the hand and rod, which were used to characterize the exploratory movements underlying task performance.

To perform the experimental task (length perception by wielding), the children sat on a chair next to an opaque black curtain positioned on their right side. Their hand was put through a slit in the curtain until the forearm was comfortably resting on an armrest, with the wrist a few centimeters beyond its edge to allow free movement. An experimenter instructed participants to maintain full grip of the rods at all times and move it using the wrist joint. Participants provided length reports, in the form of magnitude productions, by aligning the visible marker to the perceived position of the tip of the rod using a string and pulley system. This system was positioned on a 2 meters table such that it was easily reachable with their left hand. A tape measure (not visible to participants) was placed end-to-end on the stick so that the experimenter could quantify participants’ perceived length (*P*_*L*_−cm; Fig 1).

Two wooden rods with different lengths were used in the task. The mass distribution of each rod was manipulated by attaching a steel cylinder at positions corresponding to 50%, 70%, and 90% of its length. By crossing the two different lengths with three mass positions, six rods were created differing in the magnitude of their *I*_1_, computed with standard methodology, using the wrist as the axis of rotation [12]. Table 2 provides details about the characteristics of the rods. Inertial Length (*L*_I_) is the expected length report for each rod assuming appropriate attunement to *I*_1_. To compute *L*_I_ of our experimental rods (Table 2), we employed the power function relating the length of homogenous rods and *I*_1_ [Length = a (*I*_1_)^b^] [12,36]. We estimated the values of *a* (.309) and *b* (.333) using the values of length and *I*_1_ of a series of eight homogeneous rods with diameter and density equal to the experimental rods.

**Table 2:** Characteristics of the rods: Diameter (m); density (kg/m^3^); length (m); mass position (%); values of maximum principal moment of inertia—*I*_1_ (Kg/m^2^), and inertial length—*L*_I_ (m).

Rod	Diameter	Density	Length	Mass position	*I*_1_	*L*_I_
1	0.012	9.81	0.3	50	.0016	0.36
2	0.012	9.81	0.3	70	.0028	0.44
3	0.012	9.81	0.3	90	.0042	0.50
4	0.012	9.81	0.5	50	.0047	0.52
5	0.012	9.81	0.5	70	.0077	0.61
6	0.012	9.81	0.5	90	.0118	0.71

The mass distribution of each rod was manipulated by attaching a stainless steel cylinder with mass of 49.91 g.

On each experimental trial, a rod was positioned in the child’s hand who was reminded to grasp it firmly and move it around the wrist while adjusting the visible marker until he or she felt it was aligned with the tip of the rod. When the child indicated he or she was done, the experimenter recorded *P*_*L*_ and moved the marker back to its initial position. Each rod was presented three times, in random order, yielding a total of 18 trials. To characterize the wielding dynamics, the position of the hand and the position of the rod over time were recorded at a sampling frequency of 100 Hz using the Qualisys motion capture system (Qualisys Inc, Gothenburg, Sweden) with four properly calibrated ProReflex cameras. The cameras monitored the position of two infrared reflective markers placed (1) on the distal extremity of the rod and (2) on the right hand of the child (the anatomical snuffbox region; Fig 1). In each trial, recordings started when the child was given full control of the rod and told to start exploring and finished when he or she indicated the task was completed. Participants were allowed as much time as needed for each trial and could request a break if they felt tired.

Several procedures were used to familiarize the children with the task. Children were given three rods (not used during the experiment) and were instructed on how to use the pulley system to indicate their length, with and without vision. The experimental trials began only after children demonstrated clear understanding of the restrictions on the exploratory procedure (e.g., did not raise the forearm from the armrest during exploration; move the rod through the wrist only), and followed task instructions (e.g., matched the marker on the pulley system with the tip of the rod when visual information about the rod was available). We told children that the rods used for testing would be different from the ones used during practice.

### Data analyses

#### Haptic perceptual performance

The mean of the three length reports provided for each rod was computed to obtain a measure of *P*_*L*_. We computed the percent average deviation (AD%) of the three reports provided for each rod from *P*_*L*_ and percent root mean square error (RMSE%) as measures of reliability and accuracy, respectively (see [37]). The latter measure expresses the average deviations of length reports from *L*_I_, or the expected length given *I*_1_.

#### Wielding dynamics

**Fig 2:**
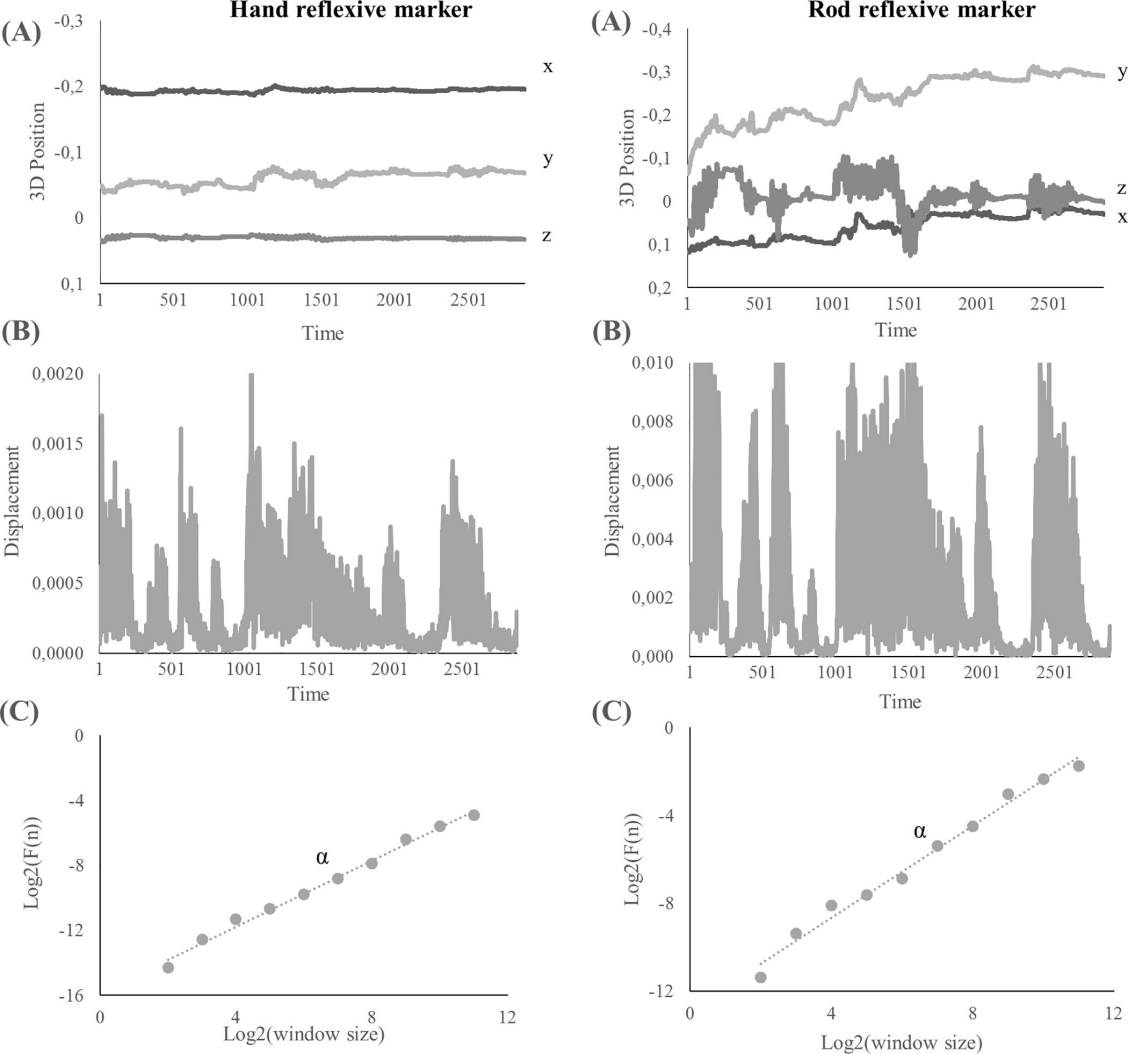
Estimate the fractality of wielding dynamics in each trial of the reflective markers positioned on the hand (left) and the rod (right). **(A)** 3D position time-series of the hand and rod recorded during wielding (sampling rate = 100Hz). **(B)** Time-series of Euclidean displacements from the 3D position time-series of the hand and rod **(C)** Detrended Fluctuation Analysis (DFA) to estimate trial-by-trial alpha reflecting the relationship between magnitude of fluctuation and time-scale.

We characterized wielding dynamics as temporal correlations (or fractality) in the detrended fluctuations in the exploratory movements of the hand and rod. Twenty-one participants in each of the two groups completed 18 trials generating a total of 756 recordings of three-dimensional positions of hand and rod over time. We computed time-series of three-dimensional hand and rod displacements by calculating the Euclidian distance between successive samples in the hand and rod position time-series, respectively (Fig 2). The mean length of the resulting time-series was 1952.77 data points (*SE* = 1320.76).

To estimate the fractality of wielding dynamics, the 756 displacement time-series of the hand and rod were submitted to Detrended Fluctuation Analysis (DFA [38]; Fig 2). DFA estimates the association between amount of root-mean-square (RMS) variation in a signal (the displacement time-series) with the timescales of measurement. The first step of this analysis is to integrate the original signal, x(t), of length N, by computing the successive cumulative sums of difference scores. This procedure produces a new series *y*(*t*):

$$y(t)=\sum_{i=1}^{N}x(i)-\bar{x(t)},$$ (1)

where $\bar{x(t)}$ is the time-series mean. The second step is to fit linear trends, *y_n_*(*t*), to nonoverlapping windows of length n (e.g., 8, 16, 32, 64, etc.) and compute the RMS over each fit. RMS is an average of the residuals around the linear fit and, therefore, captures the magnitude of fluctuation in the signal at the various window sizes. The RMS over each window size (with n < N/4) is described by the following fluctuation function *F*(n):

$$F(n)=\sqrt{(\frac{1}{N})\sum_{i=1}^{N}{\left[y(t)-y_{n}(t)\right]}^{2}}$$ (2)

The third step is to plot *F*(n), that is, plot RMS against window size (DFA plot). On standard scales, *F*(n) is a power law. Therefore, in log-log coordinates, a linear trend is observed. The fourth and final step is to compute the slope α of a regression line fitting the DFA plot. The magnitude of α provides an estimate of the temporal correlations or variability structure in the time-series: α ≈ 0.5 indicates no temporal correlations or random pattern (i.e., white noise); α ≈ 1 indicates long-range correlations or persistent structure (i.e., fractal, 1/f, or pink noise); and α ≈ 1.5 indicates highly persistent structure (i.e., Brownian motion; [39–41]).

## Results and discussion

### Dynamic touch performance

**Fig 3:**
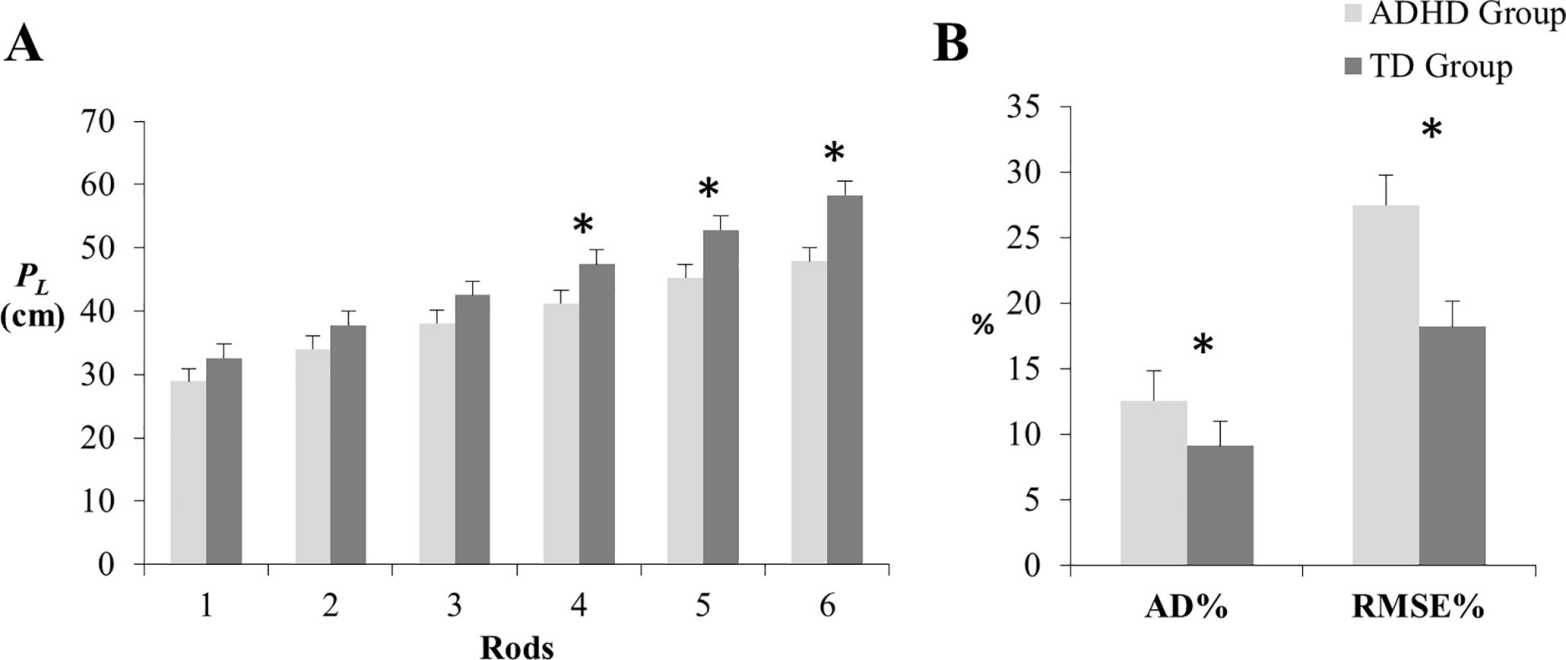
**(A)** Magnitude of the group difference in perceived length (*P*_*L*_) as a function rods and **(B)** Magnitude of the group difference in percent average deviation (AD%) and percent root mean square error (RMSE%). *Asterisks* indicate significant difference (*p* < 0.05). *Vertical lines* indicate standard error. ADHD Group: Attention-deficit hyperactivity disorder; TD Group: Typical development.

To determine whether perceptual performance is affected in children with ADHD, *P*_*L*_ was submitted to an Analysis of Variance (ANOVA) with a between subjects’ factor (Group: ADHD vs TD) and a within subjects’ factor (Rod: six levels of *I*_1_). The analysis revealed a significant main effect of Group, *F*(1, 40) = 8.50, *p* = .006; $\eta_{p}^{2}$ = .175, with children in the ADHD group showing shorter *P*_*L*_ (M = 39.08; SD = 10.67) than children in the TD group (*M* = 45.25, *SD* = 11.65). There was also a main effect of Rod, *F*(5, 200) = 127.44, *p* < . 0001; $\eta_{p}^{2}$ = .761. Linear contrast indicated a significant increase in *P*_*L*_ as a function of *I*_1_, p < .0001; $\eta_{p}^{2}$ = .860. Finally, there was a significant Group x Rod interaction, *F*(5, 200) = 2.81, p = .041; $\eta_{p}^{2}$ = .066. Simple effect analyses showed that differences between groups were statistically significant only for rods 4, *F*(1, 40) = 5.39, *p* = .025; 5, *F*(1, 40) = 7.07, *p* = .011; and 6, *F*(1, 40) = 12.04, *p* = .001 (Fig 3).

To determine whether differences in performance were indicative of ADHD-related deficits in dynamic touch, AD% and RMSE% were compared between groups. Mann-Whitney tests were employed because both variables were not normally distributed. Results indicated differences between groups in both AD% (*U* = 129, *p* = .021) and RMSE% (*U* = 111, *p* = .006) (Fig 3). As smaller values of AD% and RMSE% indicate greater reliability and accuracy, respectively, children in the TD group had significantly better reliability and accuracy of *P*_*L*_ than children in ADHD group, confirming our first hypothesis.

### Dynamic touch in ADHD: Wielding dynamics moderate information use

The scaling exponents estimated for the displacement time series of the rod (α_rod_) and for the displacement time series of the hand (α_hand_) are consistent with fractal fluctuations in both groups [ADHD: *M*α_rod_ = 1.1 (*SD* = .11), *M*α_hand_ = 1.07 (*SD* = .10); TD: *M*α_rod_ = 1.09 (*SD* = .23), *M*α_hand_ = 1.05 (*SD* = .09)]. We used linear mixed effect regression model to estimate the contributions of *I*_1_, wielding dynamics (fractal scaling of hand and rod), Group (ADHD = 0, Typical = 1), and the interactions between these factors to trial-by-trial variations in length reports. Our model also included trial number to account for any changes in length reports due to experience with the task. All lower and higher order interactions were included.

The expected collinearity between α_rod_ and α_hand_ (*r* = 0.65, *p* < 0.05) recommended using Principal Component Analysis such that both variables could figure in the same stable model. This analysis generated two orthogonal variables (PC_1_ & PC_2_), each capturing the same proportion of total variance in the original variables. The linear combination representing PC_1_ (PC_1_ = 0.95* α_rod_ + 0.31* α_hand_) indicates that its score is primarily affected by and change in direct proportion to α_rod_. In contrast, the linear combination representing PC_2_ (PC_2_ = -0.31* α_rod_ + 0.95 α_hand_) indicates that its score is primarily affected by and change in direct proportion to α_hand_ (note the similar weighing). Thus, for the sake of clarity, we will hereafter refer to PC_1_ as PC_rod_ and to PC_2_ as PC_hand_. The effect of adding predictors required comparison of deviance between model and data (-2 Log Likelihood or -2LL) between a larger model and a simpler nested model. Change in -2LL is distributed as chi-square with degrees of freedom equal to the difference in the number of parameters between nested models allowing test of statistical significance. Only interactions that significantly improved fit were kept in the final model.

Our final model included the following interactions: PC_rod_ × PC_hand_ × Trial, PC_rod_ × *I*_1_ × Group, PC_hand_ × *I*_1_ × Group as well as all component lower-order interactions and main effects (Table 3). All potential higher order interactions linking these two families of interactions failed to improve model fit, suggesting that the interaction PC_rod_ × PC_hand_ × Trial was not modified by *I*_1_ or Group. This model generates predictions that correlate well with the actual trial-by-trial length reports, *r* = .82, *p* < .0001, indicating that the model explains roughly 67.1% of the observed variability in the length reports provided by participants.

**Table 3:** Linear mixed effect regression model for length reports.

Predictor	B	SE	p value
Intercept	30.05	1.62	< .0001
*I*_1_	1886.36	134.48	< .0001
PC_hand_	-61.91	15.58	< .0001
PC_rod_	12.24	9.320	.19
PC_rod_ × PC_hand_	-93.58	35.62	< .01
PC_rod_ × Trial	-.3997	.4300	.34
PC_hand_ × Trial	2.436	.8224	< .01
PC_rod_ × PC_hand_ × Trial	62.846	2.678	< .05
*I*_1_× Group	513.28	182.92	< .01
PC_hand_ × *I*_1_× Group	-5138.99	2398.04	< .05
PC_rod_ ×I_1_ × Group	3494.03	1285.99	< .01
PC_hand_ × *I*_1_	5025.08	1742.40	< .01
PC_rod_ × *I*_1_	-2865.49	1157.78	< .05
Trial	-.0424	.0616	.50
Group	2.258	2.253	.32
PC_rod_ × Group	-5.927	9.861	.55
PC_hand_ × Group	3.228	1.766	.07

B = coefficient; SE = standard error of coefficient estimation.

#### General effects

Results of the model replicated usual findings in dynamic touch research: length reports were related to (a) *I*_1_ and (b) wielding dynamics, regardless of diagnosis. The model advances current research on dynamic touch because it captures the specific contributions of hand and rod’s movement fractality to length reports. Results show a significant main effect of PC_hand_, such that higher hand’s movement fractality was related to shorter length reports. It is possible that the relationship between hand exploratory movement fractality and length reports is mediated by the mechanical properties of the rods. Specifically, greater fractality of hand’s movement can be expected with rods of lower *I*_1_, which are tied to shorter *P*_*L*_. This interpretation is supported by research showing that (a) hand-held objects with lower *I*_1_ impose weaker constraints on exploratory movement [42], and (b) these weaker constraints allow fluctuations to distribute over a wider range of time scales and so to embody stronger fractal structure [43–46].

No main effect of PC_rod_ on length reports was observed. However, rod’s movement fractality magnifies the effect of hand’s movement fractality on length reports when both change in the same direction (PC_rod_ x PC_hand_), in particular at earlier trials (PC_rod_ x PC_hand_ x Trial). It is not clear at the moment what might explain the “magnifying” effect of rod’s movement fractality nor why it decreases with repeated trials. However, the significant interaction effects of *I*_1_ with both PC_rod_ and PC_hand_ suggests that both rod and hand’s movement fractality are required to account for the use of mechanical information during dynamic touch tasks. Importantly for present purposes, the particular wielding dynamics used to modify the emphasis on *I*_1_ differed between ADHD and TD groups (PC_hand_ x *I*_1_ x Group and PC_rod_ x *I*_1_ x Group) and will be addressed in more detail in turn.

#### Group-specific effects

The effect of *I*_1_ on length reports was significantly different between groups (*I*_1_ x Group), suggesting distinct scaling relations between the informational variable and the to-be-perceived property (i.e., different calibration). Children in the TD group changed their perceptual reports more than children with ADHD as a function of changes in *I*_1_. As noted, the effect of *I*_1_ on length reports was modified by wielding dynamics in different ways by children with and without ADHD. For TD children, the effect of *I*_1_ was emphasized when PC_hand_ was lower (*I*_1_ x PC_hand_ x Group) and PC_rod_ was higher (*I*_1_ x PC_rod_ x Group) and for children with ADHD, the effect of *I*_1_ was emphasized when PC_hand_ was higher (*I*_1_ x PC_hand_) and PC_rod_ was lower (*I*_1_ x PC_rod_). In conjunction, these results support our second hypothesis and will be addressed in more details in the general discussion.

## General discussion

The present study provides empirical evidence that dynamic touch is affected in children with ADHD adding to the growing literature about perceptual deficits in this population (cf. [47] for review). Results reinforce the need to study the implications of perceptual deficits in general and dynamic touch in particular to the challenging functional problems presented by children with ADHD. Children with ADHD underestimated the magnitudes of rod length, in particular for longer rods (Fig 3). Notably, a similar pattern of performance was demonstrated by children with DCD [20]. ADHD and DCD are developmental disorders that present common characteristics such as difficulty in tasks that require manipulation of objects [4,48]. Therefore, deficits in dynamic touch may be at the basis of difficulties in manual skills presented by children with these (commonly co-occurring) medical conditions.

One might interpret the underestimation of object length observed in children with ADHD and DCD as signaling a generally reduced receptivity of mechanical stimulation. This interpretation would be consistent with evidence of lower activation of neural areas related to somatosensory perception [49–51] (cf. [48]). However, if deficits in dynamic touch were a simple function of impaired neural receptivity, underestimation would be similar across rods or, at best, differences in performance would be reduced for rods with higher *I*_1_ that provide relatively greater magnitudes of mechanical stimulation. The opposite was true for both children with ADHD (present study) and DCD [20]. Thus, this pattern of result would be consistent with differences in *how* mechanical information supporting dynamic touch is used.

The results of our regression model indeed support this assertion: while children in both groups (ADHD and TD) increased length reports with increases in *I*_1_, changes in perceived length associated with changes in *I*_1_ were less pronounced in children with ADHD (Table 3). The lower emphasis on *I*_1_ can explain their lower accuracy and reliability in the task when compared to TD children (Fig 3). Together, these results suggest that children with ADHD were sensitive to trial-by-trial variations in the value of *I*_1_, but were relatively less sensitive to information about the appropriate scaling of *I*_1_ to length that becomes available over longer time scales. In other words, these atypical developmental trajectories leave intact those mechanisms supporting information detection (i.e., the more or less instantaneous pick up of information) and rather affect calibration mechanisms refining use of information over time.

We hypothesized that the dynamics of exploratory movements predict differences in the use of information by children with ADHD when compared to their TD peers. Results supported this hypothesis. There were significant differences between groups in *how* the fractality of exploratory movements of the rod and hand moderated children’s emphasis on *I*_1_. In TD children, lower hand fractality and greater rod fractality was related to increased emphasis on *I*_1_ (Table 3). In contrast, children with ADHD increased emphasis on *I*_1_ when the exploratory movement was characterized by greater hand fractality and lower rod fractality (Table 3).

It is not fully clear at this point what underlies the particular differences between groups in how fractality was related to the use of *I*_1_. However, previous studies that investigated the effect of rod vibration on dynamic touch performance provide grounds for an initial speculation [32,33]. Results showed an increased emphasis on *I*_1_ over time associated with striking, adding to recent results implicating the role of rod vibrations in perceptual calibration even when instructions to participants only entail firm grasp and wielding [32,33]. As noted, when lower fractality of the hand’s exploratory movement co-occurred with high fractality of the rod’s movement, sensitivity to *I*_1_ increased in TD children. This result may indicate that when TD children constrained hand motions during exploration so as to enhance stimulation by richly structured rod vibrations within the hand, their perceptual performance became more tightly coupled to the relevant informational parameter, possibly through a process of self-guided calibration.

Children with ADHD commonly present excessive, mildly disorganized overt motor activity and, thus, are less prone to constrain hand motions and direct their processes of stimulation. It is possible, nonetheless, that in trials where children with ADHD demonstrated higher movement fractality, the less ordered overt motions produced by children with ADHD resulted in a general increase in perceptual sensitivity. In particular, greater fractality in hand motions suggests stronger interactions of overt exploratory activity across scales, which can be expected to promote more efficient information sampling and, thereby, explain the increased emphasis on *I*_1_ (e.g., see [41] for a discussion of fractal systems as “space filling”). The implication is that, with the right level of fractality, the seemingly irrelevant motor activity of children with ADHD might play a non-obviously functional role.

A potential functional role of hyperactivity has indeed been previously demonstrated. For example, Sarver et al. [52] have shown that the level of performance in a memory task by children with ADHD was directly proportional to the degree of unrelated motor activity they produced during task performance. It is possible that the heightened motor activity enhanced multi-scale fluctuations that increase perceptual awareness of the environment, freeing up resources for the cognitive task. If this hypothesis is confirmed, cognitive-behavioral interventions designed to directly reduce excessive movements should be reconsidered ([53–55]). At a larger scale, beyond the specific scale of ADHD research, converging lines of research in movement science and in biological sciences more generally are pointing increasingly towards clearer appreciation that higher-dimensional, “noisier,” more random or stochastic processes are a key part of healthy developmental trajectories [56–59]. The notion of noise supporting biological order is not exactly new [60], but it can be “nonobvious” or seem “unrelated” to researchers not knowing to seek evidence of its role [61], and that evidence can go entirely undetected with modeling that does not make it explicit [62]. The present work is the latest example of how motor variability can be a window on the development of individual differences in perceptual-motor response—a window made clearer in some cases by fractal modeling [25,63,64].

An immediate question for clinical applications of this insight might be: So, if “noisy” movements can support developmental trajectories, can we leverage endogenous fluctuations with unobtrusive noise-based interventions and therapies? The short answer seems to be “yes.” Noise-based stimulation below sensory threshold can make both posture and gait more stable when applied to the plantar surface of the foot [65–67] and can make breathing more regular when applied to an infant’s mattress [68,69]. These noise-based stimulations appear to work better for individuals with greater apparent pathology or instability [70,71]. Perhaps, noise-based interventions may provide an unobtrusive but fruitful way to facilitate functional performance of children with ADHD [72].

### Conclusion

The present study showed that children with ADHD have deficits in dynamic touch that may be crucial for the organization of effective, task and context sensitive movement patterns. These deficits were manifested as reduced sensitivity to mechanical information that supports perception of object properties by wielding. Importantly, differences in haptic perceptual performance between children with and without ADHD were related to the temporal correlations (degree of fractality) observed in the subtle, seemingly irrelevant fluctuations of exploratory movements. In general lines, this result can be taken to reflect differences in the coordination of multiscale processes involved in information sampling and use (e.g., coordination of the moment-to-moment pick up of information with the longer time-scale processes that support appropriate calibration). Future studies using a learning paradigm can be designed to better gauge the adaptability of perceptual performance of children with ADHD when explicit knowledge of results is provided over time.
